# Antitumour Organometallics. III. *In Vivo* Activity of Diphenylantimony(III) and Diorganotin(IV) Dithiophosphorus Derivatives Against P388 Leukemia

**DOI:** 10.1155/MBD.1994.73

**Published:** 1994

**Authors:** Bernhard K. Keppler, Cristian Silvestru, Ionel Haiduc

**Affiliations:** 1 Anorganisch-Chemisches Institut der Universität Heidelberg In Neuenheimer Feld 270 Heidelberg 1 D-6900 Germany; 2 Facultatea de Chimie Universitatea "Babes-Bolyai" Cluj-Napoca RO-3400 Romania

## Abstract

Diphenylantimony(III) and diorganotin(IV) derivatives of dithiophosphorus ligands, *i.e. *Ph_2_SbS_2_PR′_2_ (R′ = Ph, OPr-i) and R_2_Sn(S_2_PR′_2_)_2_ (R =  n-Bu, Ph, R′ = Ph; R =  Ph, R′ = OPr-i), have been screened against P388 leukemia in mice. All the compounds showed marginal activity towards this tumor system, some of them increasing the life span of the animals with more than 20%. The best results were obtained with (di-iso-propylphosphorodithioato)diphenylantimony(III) which exhibited a T/C value of 136%, at a dose of 5 mg/kg, administered on days 1,2 and 3 after tumor transplantation.


### ANTITUMOUR ORGANOMETALLICS. III. *IN VIVO* ACTIVITY OF DIPHENYLANTIMONY(III) AND DIORGANOTIN(IV) DITHIOPHOSPHORUS DERIVATIVES AGAINST P388 LEUKEMIA

Bernhard K. Keppler

Anorganisch-Chemisches Institut der Universität Heidelberg, In Neuenheimer Feld 270, D-6900 Heidelberg 1, Germany

Cristian Silvestru and Ionel Haiduc

Facultatea de Chimie, Universitatea "Babes-Bolyai", RO-3400 Cluj-Napoca, Romania

### ABSTRACT

Diphenylantimony(III) and diorganotin(IV) derivatives of dithiophosphorus ligands, *i.e.* $Ph_2SbS_2PR'_2$ (R' = Ph, OPr-i) and $R_2Sn(S_2PR'_2)_2$ (R = n-Bu, Ph, R' = Ph; R = Ph, R' = OPr-i), have been screened against P388 leukemia in mice. All the compounds showed marginal activity towards this tumor system, some of them increasing the life span of the animals with more than 20%. The best results were obtained with (di-iso-propylphosphorodithioato)diphenylantimony(III) which exhibited a T/C value of 136%, at a dose of 5 mg/kg, administered on days 1, 2 and 3 after tumor transplantation.

### INTRODUCTION

In recent years, the potential antitumor activity of organometallics, *i.e.* compounds containing direct **metal-carbon** bonds, has received an increased attention,[1] since more and more derivatives of either transition and Main Group metals were found to exhibit interesting inhibitory properties on animal tumor systems. Among Main Group metal compounds, organotin(IV) derivatives occupy a top position related to their antitumor effects, with some of them being even more active than cisplatin in *in vitro* tests.[2-9]

Interest in organoantimony(III) compounds as potential antitumor agents arose in recent years when diphenylantimony(III) derivatives of dithiophosphorus ligands were reported as the first organoantimony compounds to exhibit antitumor properties *in vitro* and *in vivo* against Ehrlich ascites tumor.[10-12] Comparative studies in this mouse tumor system, using diphenylantimony(III) and diphenyltin(IV) compounds containing the same dithiophosphorus ligands, pointed out that organometallic di-iso-propylphosphorodithioates were more active than diphenylphosphinodithioato analogues, and the organoantimony derivatives were more active than organotins. Moreover, $Ph_2SbS_2P(OPr\text{-}i)_2$ (5 mg/kg/day, i.p. on days 1, 3 and 5) produced an increase in lifespan of 83% and a cure rate of 30% in mice bearing this tumor.[10]

Here we report the results obtained using the same compounds as above, on P388 leukemia in mice. Additionally, a dibutyltin(IV) derivative, *i.e.* $n\text{-}Bu_2Sn(S_2PPh_2)_2$, was included in the screening, since a lot of previous reports concerning the antitumor activity of organotins[5,8,9] have suggested that the presence of the di-n-butyltin moiety improve the *in vitro* inhibitory effects against human tumor cell lines (MCF-7 and WiDr) in comparison to analogous compounds containing phenyl groups bound to tin.

### MATERIALS AND METHODS

*Animals.* The DBA/2 (female, *ca.* 20 g) and $BDF_1$ mice (female, 20-22 g) were provided by Zentralinstitut für Versuchstierkunde, Hannover (FRG) and kept under conventional conditions: 3 mice per Macrolon III cage, tap water and Altromin pellets *ad libitum.* Room temperature was at about 20°C; room air kept circulating 20 times/hour; a light/dark rhythm was maintained over 12 hours.

*Compounds.* The organometallic compounds used as antitumor agents were prepared and purified as described earlier: bis(diphenylphosphinodithioato)diphenyltin(IV), $Ph_2Sn(S_2PPh_2)_2$ (compound **1** - KP 1215),[13] bis(di-iso-propylphosphorodithioato)diphenyltin(IV), $Ph_2Sn[S_2P(OPr\text{-}i)_2]_2$ (compound **2** - KP 1216),[14] bis(diphenylphosphinodithioato)di-n-butyl-tin(IV), $n\text{-}Bu_2Sn(S_2PPh_2)_2$ (compound **3** - KP 1217),[13] (di-iso-propylphosphorodithioato)-diphenylantimony(III), $Ph_2SbS_2P(OPr\text{-}i)_2$ (compound **4** - KP 1218),[15] and (diphenylphosphino-dithioato)diphenylantimony(III), $Ph_2SbS_2PPh_2$ (compound **5** - KP 1219).[16]

*In vivo experiments.* P388 leukemia cells were implanted intraperitoneally into DBA/2 mice for propagation 7 days before the experiment. The tumor cells were taken from these animals at the begining of the experiment immediatelly after cervical dislocation. Then we implanted $10^6$ of these cells, suspended in 0.2 ml of physiological saline, intraperitoneally into female $BDF_1$ mice, body weight 20-22 g, for testing. Then the mice were arbitrarily divided into groups of each time six animals, with one group serving as control. Therapy started 24 hours *post transplantationem* (= day 1) with a single dose of the respective organometallic compounds, applied intraperitoneally as suspension in physiological saline. As emulsifiers we used cremophorEL/propylenglycole. Therapy was repeated on days 2 and 3.

### RESULTS AND DISCUSSION

The organometallic compounds used in this screening differ not only in the nature of the metal, organic groups bound to the metal atom, presence of aryl or alkoxy groups attached to phosphorus, but also by the molecular structure in solid state (Figure 1). Thus, all three diorganotin(IV) derivatives are monomeric compounds. However, for diphenyl- and di-n-butyltin(IV) diphenylphosphinodithioates **1** and **3**, the infrared and Mössbauer data suggest an angular orientation of the Sn-C bonds and anisobidentate coordination of the dithioligands,[13] while for the di-iso-propylphosphorodithioato analogue **2**, the C-Sn-C angle is 180° and the ligands are isobidentate as determined by X-ray diffractometry.[14] For both diphenylantimony(III) derivatives **4** and **5**, the solid state structures were also investigated by the X-ray method. The phosphorodithioato **4** has a polymeric chain structure developed through weak Sb...S secondary bonds, the ligand acting effectivelly as a triconnective moiety.[15] The dithioligand has also a triconnective behavior in the phosphinodithioato analogue **5**, but leading in this case to distinct dimeric units[16] (Figure 1).

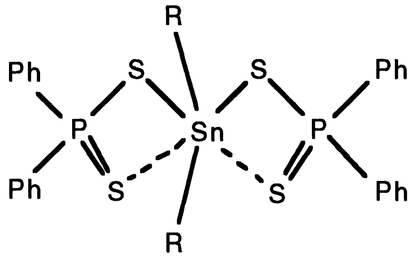

**1**, R=Ph
**3**, R=Bu-n

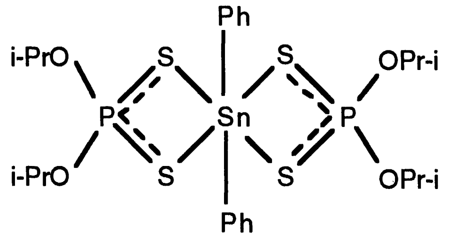

**2**

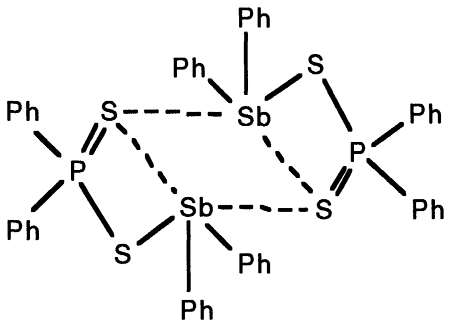

**5**

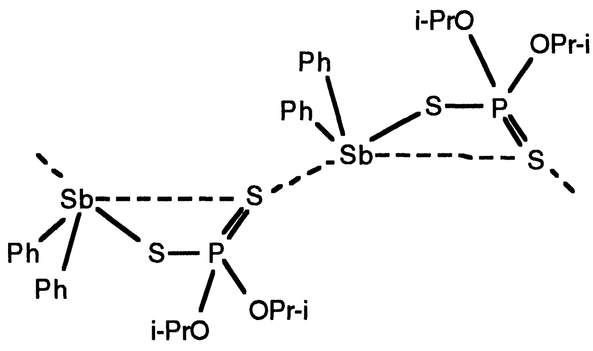

**4**

**Figure 1.** Molecular structures of organoantimony(III) and organotin(IV) compounds tested as antitumor agents against P388 leukemia in mice.

The antitumor effects of the above organometallic compounds against P388 leukemia in mice are listed in Table 1. All the diorganotin(IV) derivatives **1**, **2** and **3**, and the (diphenylphosphinodithioato)diphenylantimony(III) **5**, exhibited only marginal activity (T/C *ca.* 120%). Although the T/C value of 127% obtained for compound **3** was the best in the series of organotins, the presence of the di-n-butyltin(IV) moiety did not spectacularly improve the antitumor properties.

As in the previous experiments on Ehrlich ascites tumor,[10-12] the most active compound was (di-iso-propylphosphorodithioato)diphenylantimony(III). It exhibited a T/C of 136% at a dose of 5 mg/kg, administered on days 1, 2 and 3, after tumor transplantation. However, when increasing the dose, the toxic effect of this compound seems to be stronger, since a decreased T/C value (*i.e.* 118%) was obtained.

**Table 1.** Test results of organoantimony(III) and organotin(IV) compounds against P388 leukemia in mice.

| Compound | Day of death | Dose[a] | | T/C[b] |
|---|---|---|---|---|
| | | mmol/kg | mg/kg | (%) |
| Control | 11, 11, 11 | - | - | 100 |
| Cisplatin | 20, 21, 27, 27, 33, 33 | 0.013 | 4 | 245 |
| **1** | 3, 13, 13, 14, 16, 16 | 0.006 | 5 | 123 |
| **2** | 12, 12, 13, 13, 13, 13 | 0.007 | 5 | 118 |
| **3** | 11, 12, 14 14, 15, 16 | 0.0065 | 5 | 127 |
| **4** | 12, 12, 15 15, 15, 26 | 0.01 | 5 | 136 |
| | 12, 13, 13, 13, 14, 14 | 0.02 | 10 | 118 |
| **5** | 12, 12, 13, 13, 13, 14 | 0.0095 | 5 | 118 |
| | 11, 12, 13, 14, 16, 16 | 0.019 | 10 | 123 |

[a] Therapy was carried out on days 1, 2 and 3, after tumor transplantation; [b] T/C (%) = (median survival time of treated animals *vs.* median survival time of control animals)x100.

### REFERENCES

1. I. Haiduc and C. Silvestru, *Organometallics in Cancer Chemotherapy*, CRC Press, Boca Raton, Florida; Vol.I., 1989; Vol.II., 1990.
2. M. Gielen (Ed.), *Tin-Based Antitumor Drugs*, NATO ASI Series: Cell Biology, Vol.37, Springer Verlag, Berlin, 1990.

3. M. Bouâlam, R. Willem, M. Biesemans, B. Mahieu and M. Gielen, *Heteroatom Chem.*, 1991, **2**, 447.
4. M. Bouâlam, R. Willem, M. Biesemans and M. Gielen, *Appl.Organomet.Chem.*, 1991, **5**, 497.
5. S.W. Ng, V.C. Kumar Das, M. Gielen and E.R.T. Tiekink, *Appl.Organomet.Chem.*, 1992, **6**, 19.
6. M. Gielen, J. Meunier-Piret, M. Biesemans, R. Willem and A. El Khloufi, *Appl.Organomet. Chem.*, 1992, **6**, 59.
7. M. Gielen, P. Lelieveld, D. de Vos, H. Pan, R. Willem, M. Biesemans and H.H. Fiebig, *Inorg.Chim.Acta*, 1992, **196**, 115.
8. M. Gielen, A. El Khloufi, M. Biesemans and R. Willem, *Polyhedron*, 1992, **15**, 1861.
9. M. Gielen and R. Willem, *Anticancer Res.*, 1992, **12**, 257.
10. C. Silvestru, C. Socaciu, A. Bara and I. Haiduc, *Anticancer Res.*, 1990, **10**, 803.
11. A. Bara, C. Socaciu, C. Silvestru and I. Haiduc, *Anticancer Res.*, 1991, **11**, 1651.
12. C. Socaciu, A. Bara, C. Silvestru and I. Haiduc, *In Vivo*, 1991, **5**, 425.
13. C. Silvestru, F. Ilies, I. Haiduc, M. Gielen and J.J. Zuckerman, *J.Organomet.Chem.*, 1987, **330**, 315.
14. H. Lefferts, K.C. Molloy, J.J. Zuckerman, I. Haiduc, M. Curtui, C. Guta and D. Ruse, *Inorg. Chem.*, 1980,**19**, 2861.
15. C. Silvestru, M. Curtui, I. Haiduc, M.J. Begley and D.B. Sowerby, *J.Organomet.Chem.*, 1992, **426**, 49.
16. C. Silvestru, L. Silaghi-Dumitrescu, I. Haiduc, M.J. Begley, M. Nunn and D.B. Sowerby, *J.Chem.Soc., Dalton Trans.*, 1986, 1031.

**Received: August 18, 1993 - Accepted: September 9, 1993**

